# The fascial-interstitial system and the sanjiao-mocou system: an analogy-based hypothesis for the anatomical basis of meridian pathways

**DOI:** 10.3389/fphys.2026.1795656

**Published:** 2026-03-27

**Authors:** Yu Xia, Yuxin Jia, Hui Qu, Qianxue Wei, Limei Yan, Xiuqin Li

**Affiliations:** 1 Department of Obstetrics and Gynecology, Shengjing Hospital of China Medical University, Shenyang, China; 2 Ultrasound Department, Shengjing Hospital of China Medical University, Shenyang, China

**Keywords:** anatomy, connective tissue, fascial-interstitial system, integrative medicine, meridians, mocou system, Sanjiao (Triple Energizer), traditional Chinese medicine (TCM)

## Abstract

**Background:**

The anatomical basis of meridian pathways remains a central challenge in modernizing Traditional Chinese Medicine (TCM). Concurrently, modern science has redefined the integrated fascial-interstitial system as a pervasive, fluid-filled cavitary organ system involved in signaling and transport. This parallel invites novel theoretical integration.

**Objective:**

This review proposes a translational hypothesis positing that the classical TCM concept of the “Sanjiao-Mocou” system is analogous to the modern fascial-interstitial system, together constituting the anatomical and functional substrate of meridians.

**Methods:**

We conducted a systematic comparative analysis of classical TCM texts describing the Sanjiao (Triple Energizer) and Mocou (interstitial spaces) and contemporary literature on the structure and function of the fascial-interstitial system. This theoretical integration focuses on their shared attributes: being ubiquitous, fluid-transporting, cavity-containing connective tissue systems that facilitate systemic communication and homeostasis.

**Findings:**

The hypothesis elucidates how the Sanjiao-Mocou system, long understood as the “passageway for Yuan-Primordial Qi and body fluids,” aligns closely with the fascial-interstitial system’s role in interstitial fluid transport, mechanotransduction, and immune surveillance. This analogy provides a coherent, testable model where meridians may be conceptualized as specialized functional channels within this pervasive cavitary organ.

**Conclusion:**

The proposed analogy bridges a foundational TCM theory with contemporary biomedical science. It offers a potential anatomical framework for understanding meridians and opens new avenues for interdisciplinary research in biomechanics, fluid dynamics, and integrative physiology, potentially advancing the understanding of both TCM and fascial science.

## 1 Introduction

In Western anatomy, the interstitium has long been regarded as a fluid-filled space between cells, while fascia is seen as a static envelope wrapping muscles and organs. Their structure and function have been studied separately. However, in 2018, Benias and his team reported for the first time in Scientific Reports that the interstitium is a continuous, three-dimensional network distributed throughout the body, composed of a collagen scaffold, a hyaluronan matrix, and dynamic fluid (Benias et al., 2018). This study redefined the interstitium as a previously unrecognized fluid-filled interstitial space distributed throughout the human body. Building on this foundation, we propose to connect it with the meridian channels in Traditional Chinese Medicine (TCM) theory responsible for the flow of Qi and blood, in order to explore the possible correspondence between the interstitium and certain aspects of the meridian system at the anatomical level.

In parallel, research in fascial science over the past two decades has revealed another perspective: fascia, as a bilayer membranous structure, envelops, separates, and connects all organs, muscles, and nerves, forming the body’s integral mechanical support and information transmission environment (Adstrum et al., 2017). Its systemic tension network distribution—the “Anatomy Trains”—closely matches the meridian pathways described in ancient TCM texts as “running deeply between the muscles, invisible from the surface.” This has led some researchers to hypothesize that fascia could represent a modern histological correlate of the meridians.

Although the “interstitium–meridian” and “fascia–meridian” hypotheses have been widely discussed in the medical community, such debates may reflect certain limitations in perspective. Could adopting a more systemic viewpoint offer new understanding? In TCM theory, the Sanjiao (Triple Energizer) is regarded as one of the six Fu-organs. Traditionally described as “the official in charge of drainage, from which waterways emerge,” it is understood as a systemic cavity and pathway involved in water metabolism, the movement of primordial qi (yuanqi), and the process of qi transformation. Its described organization and functional attributes appear to show notable similarities to the interstitial system as characterized by Benias et al. Furthermore, the multi−layered “cavity−channel” system outlined in TCM since the Huangdi Neijing (Yellow Emperor’s Inner Canon)—progressing from the Couli (interstitial spaces of the skin and muscles) and Fenrou (intermuscular spaces) to the Xigu (larger muscular grooves and joint spaces) and Moyuan (membrane source), from exterior to interior—seems to correspond in a layered manner, both anatomically and functionally, to the fascial system described in Western medicine, from superficial to deep layers.

**Figure 1:**
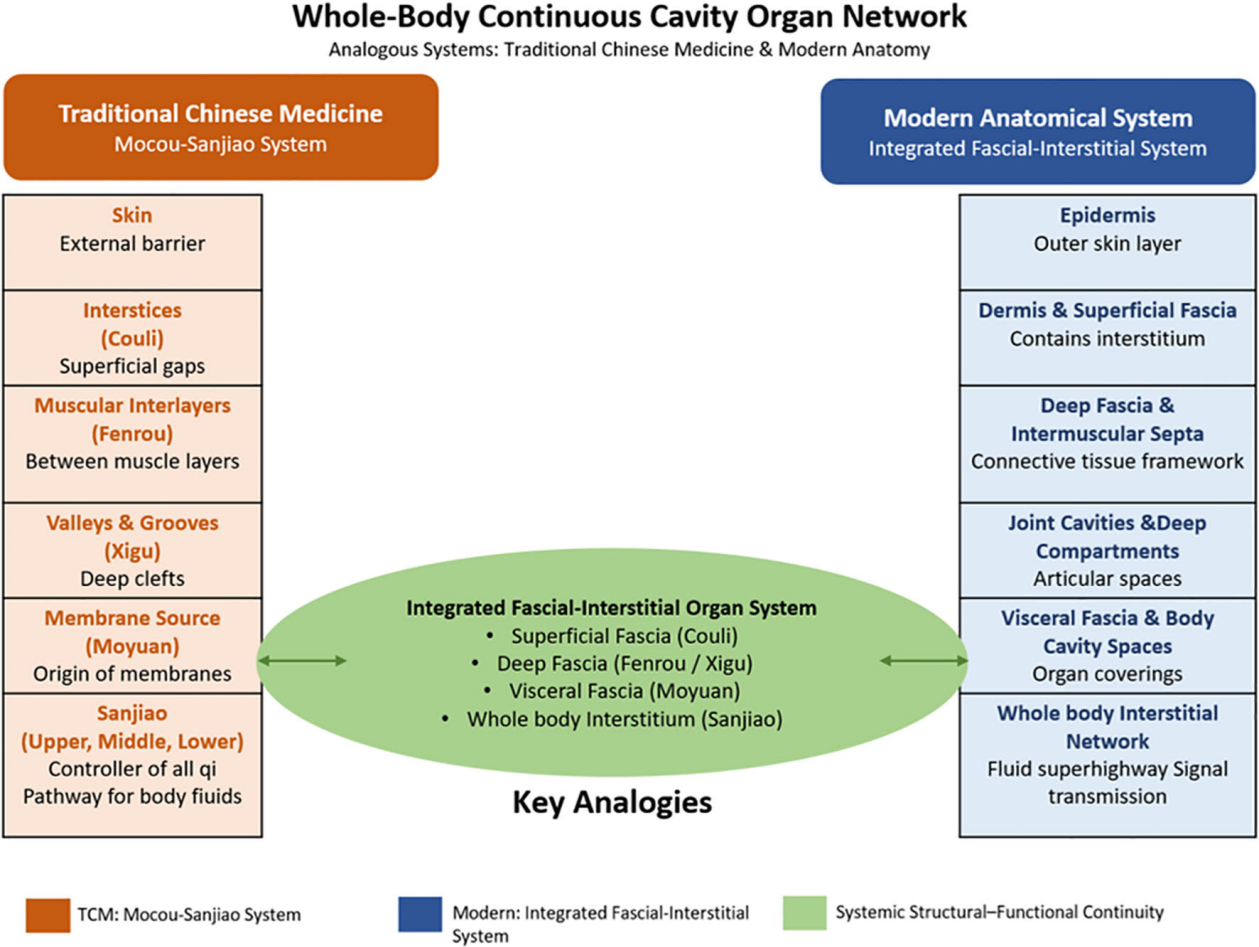
Schematic diagram illustrating the proposed analogy between the TCM “Membrane-Interstice (Sanjiao)” system and the modern integrated fascial-interstitial system. This diagram conceptualizes the integrated fascial-interstitial system as a distributed organ system (Pratt et al., 2025).

From these observations, it might be inferred that the TCM “Mocou–Sanjiao” system and the Western “fascia–interstitium” system could share certain homologous features in structure, function, and possibly developmental origin. Together, they suggest the possibility of a systemic, continuous, cavity−like organizational framework that may contribute to mechanical support, body fluid circulation, cellular communication, and energy or information transfer. This article will examine, from an interdisciplinary perspective, the analogy hypothesis between the “Mocou–Sanjiao” system and the Western “fascia–interstitium” system, considering evidence from structure to function (**Figure 1**).

The traditional separation of fascia and interstitium into distinct anatomical compartments has led to inconsistent terminology and skepticism regarding their physiological importance. A very recent comprehensive review by Pratt et al. (Stecco et al., 2025) addresses this challenge by proposing their integration into a unified “human fascial system,” conceptualized not as isolated tissues but as a distributed organ system. Building on this integrative framework, the fascial system can be defined as a layered, body-wide multiscale network of connective tissue that enables both tensional loading and shearing mobility. This system comprises four anatomical organs—superficial fascia, musculoskeletal (deep) fascia, visceral fascia, and neural fascia—each composed of variable combinations of epithelial, connective, muscle, and neural tissues. Its overarching functions arise from the contrasting biomechanical properties of two fundamental layer types distributed throughout: one predominantly collagenous and relatively stiff, the other rich in hyaluronic acid and viscous, allowing fluid flow. This unified model provides a modern anatomical foundation for conceptualizing the TCM Sanjiao-Mocou system as a continuous, body-wide cavity network for fluid and information transport.

## 2 Methods & theoretical integration

### 2.1 Terminological clarification: interstitium and fascial interstitium

In this paper, we adopt the hierarchical understanding of interstitial spaces as established in recent anatomical literature (Benias et al., 2018; Pratt et al., 2025). The term interstitium refers broadly to the continuous, body-wide network of fluid-filled spaces within tissues, which can be categorized into three interconnected levels based on scale and location: (1) fascial interstitium (10–100 μm) (Benias et al., 2018), located within fascial structures between collagen bundles; (2) perivascular/capillary interstitium (5–50 μm) (Benias et al., 2018; ND et al., 2023), surrounding blood vessels; and (3) intercellular interstitium (<1.5 μm) (Tamaki et al., 2002; Calabrese et al., 2003; Aslan et al., 2006), between adjacent cells. Each interstitial level possesses its own specific nomenclature and defining characteristics. The fascial interstitium is therefore a specific subtype of the broader interstitium, and together with the collagenous layers of fascia, it constitutes the unified fascial system as defined by Pratt et al. (2025). Just as the fascia constitutes a network of continuous structures throughout the body, the fascial interstitium likewise forms a systemic network through which water and solutes can flow. It should be particularly noted that the fascial interstitium represents the largest-scale interstitial structure in the human body, maintaining structural continuity as a whole with smaller interstitial spaces, including perivascular/capillary interstitium and intercellular interstitium (Cenaj et al., 2021; Ordner et al., 2025; Stecco et al., 2025; Theise et al., 2025). All of these interstitial spaces make up a single, body-wide network containing as much as 20% of the fluid volume of the body (Cenaj et al., 2021). This integrated system of the broad interstitium and fascial system functions as a body-wide, layered connective tissue network that provides both mechanical support (via collagenous layers) and fluid/signal transport (via hyaluronan-rich interstitial layers). This integrated system serves as the modern anatomical correlate for the TCM concepts discussed in this paper.

### 2.2 Structure and function of the interstitium: from “intercellular space” to a “systemic organ”

#### 2.2.1 Traditional view vs. modern discovery

Traditionally in Western medicine, the interstitium has been predominantly defined as a space between cells, largely understood as comprising extracellular matrix and fluid components—such as tissue fluid, lymph, and plasma. It is generally regarded as contributing to cellular support, protection, connection, and nourishment, thereby participating in the formation of a functional microenvironment. The first, the pericapillary interstitium, is a well-defined space composed of an extracellular matrix (ECM) scaffold—largely consisting of collagens—and interstitial fluid containing water and solutes (Levick, 1987; Swartz and Fleury, 2007; Kajbafzadeh et al., 2015; Stewart, 2020). A second group of smaller interstitial spaces, located between adjacent cells and continuous with the pericapillary interstitium, contain fluid but exhibit few to absent collagen fibrils (Tamaki et al., 2002; Calabrese et al., 2003; Aslan et al., 2006). The 2018 study by Benias and colleagues, however, has prompted a significant reevaluation of this conventional view. Through the use of fluorescent tracers and probe−based confocal laser endomicroscopy (pCLE) during *in vivo* endoscopic procedures, the team reported the observation of what appeared to be a dynamic, interconnected, fluid−filled “honeycomb−like” network of spaces located between collagen bundles in several human tissues, including the dermis and biliary tract (Benias et al., 2018). This interstitium stands in a complementary relationship with the collagen-rich regions distributed throughout all the body’s fasciae. Like the pericapillary spaces, these larger spaces contain water, solutes, and collagen, but they are distinguished by the presence of significant cellular components (Stecco et al., 2025).

#### 2.2.2 Proposed functions of the interstitial space network

This discovery has prompted a reconsideration of the interstitium’s function, suggesting it may extend beyond the traditional “filler” to potentially dynamic system. While it is well established that the pericapillary (perivascular) spaces surrounding microvessels facilitate the exchange of nutrients and wastes within tissues (Swartz and Fleury, 2007; Wiig and Swartz, 2012; Stewart, 2020), the discovery of the fascial interstitium has revealed further functions of the interstitial system. The functions of the interstitium can be summarized as follows. The collagen fibrous network provides structural scaffolding that determines the architecture of large interstitial spaces. It enables long-range force transmission (Wang et al., 2014) between cells across the network, serving as a potential mechanism for information transmission through the interstitium. Collagen also acts as a binding scaffold for molecules such as hyaluronic acid, elastin, and proteoglycans, many of which regulate collagen fibril self-organization and mechanics. Additionally, collagen undergoes circadian cycles of damage and repair (Yeung and Kadler, 2019), suggesting potential dynamic maintenance mechanisms within the interstitium. The extracellular matrix (ECM) of interstitial spaces exhibits several additional functions beyond facilitating fluid flow and long-range force transmission: it serves as a guiding scaffold for cell migration, directing the movement of both resident and non-resident cells, such as metastatic tumor cells and lymphocytes during inflammatory conditions (Bartolini et al., 2020); furthermore, due to the piezoelectric properties of collagen—which converts kinetic energy into electrical energy and vice versa (Minary-Jolandan and Yu, 2009)—the interstitial spaces may support electrical signal transmission independent of the nervous system, given that all fibrous tissues in the body undergo intermittent or continuous motion. According to the view of Peter Friedl and colleagues, early cancer invasion may not rely on the secretion of stroma-degrading enzymes to create a path; rather, malignant cells can exploit pre-existing interstitial channels in the body that serve as paths of least resistance (Gritsenko et al., 2012; Alexander et al., 2013; Hager et al., 2013; Gritsenko et al., 2017; Huda et al., 2018; Beunk et al., 2019). Prior studies have demonstrated that tumors can metastasize via the biliary tract interstitium and the colonic interstitium (Cenaj et al., 2021). The hyaluronan-rich environment of the interstitium suggests that hyaluronidases may play a role in facilitating cellular traversal through interstitial spaces (Lennon and Singleton, 2011; Cox, 2021). Thus, this fluid-filled interstitial space network throughout the body may have important implications for molecular signaling, cell migration, and the dissemination of both malignant and infectious diseases.

#### 2.2.3 The interstitium as an organ: a hypothesis and its context

Based on these observations, Benias et al. have proposed that this extensive network might be conceptualized as a previously overlooked independent organ, whose functional attributes invite comparison with the meridian system described in TCM (Benias et al., 2018). This modern perspective finds resonance in earlier physiological work, which has long recognized the interstitium, in coordination with the lymphatic system, as playing a crucial role in regulating extracellular fluid volume and distribution (Aukland and Reed, 1993). The conceptualization of the interstitium as a continuous fluid network thus appears to align with and extend this classical understanding, together highlighting a potentially significant systemic role for the interstitium in maintaining homeostasis.

While the hypotheses regarding the “interstitium as an organ” and its “meridian-like function” continue to be discussed—with some scholars noting the need to reconcile it with classical organ definitions or to further elucidate its relation to meridian phenomena—the findings undoubtedly offer a fresh perspective for exploring systemic physiology and therapeutic approaches. Interestingly, within TCM theory, the concept of the Sanjiao presents a functional paradigm that may bear notable parallels to the proposed characteristics of this integrated fascial-interstitial network.

### 2.3 Structure and function of the Sanjiao: the “largest cavitary organ” in TCM system theory

#### 2.3.1 Conceptual anatomy: the three Jiaos

Within the framework of TCM Zangxiang (organ manifestation) theory, the Sanjiao is regarded as one of the six Fu-organs, often noted for its distinctive characterization as having “a name but no definite form,” which suggests a systemic rather than a discrete anatomical entity. Its conceptualized anatomical domain is commonly divided into three regions: the Upper Jiao (above the diaphragm, associated with the heart and lungs), the Middle Jiao (between the diaphragm and the umbilicus, linked to the spleen, stomach, liver, and gallbladder), and the Lower Jiao (below the umbilicus, pertaining to the kidneys, bladder, and intestines).

#### 2.3.2 Functional synthesis: from waterways to Qi dynamics

Historical texts present varied emphases regarding its core functions: for instance, the Suwen (Basic Questions) describes it as “the official in charge of drainage, from which waterways emerge,” (Daihua, 2005) underscoring its perceived role in water metabolism; whereas the Nanjing (Classic of Difficult Issues) refers to it as “the special envoy of the Original Qi (Yuanqi),” (Jianhua, 2018) highlighting its conceptual pathway for the movement of Primordial Qi and the dynamics of Qi. In modern TCM synthesis, its functions are often summarized as “governing all types of Qi and overseeing the Qi transformation and water metabolism of the whole body,” positioning it as a central concept in understanding visceral coordination and the elimination of metabolic waste (Sun, 2012).

Some scholars, integrating perspectives from modern physiology, have proposed that the functions of the Sanjiao may be viewed as encompassing seven aspects: serving as a primary conduit for the circulation of body fluids (blood and jin-ye); acting as a distribution pathway for nutrients derived from food and drink; functioning as a hub for waste excretion; possessing characteristics analogous to those of the lymphatic system; having potential relations to endocrine function; exerting a governing influence on the meridian system; and serving as a source and site of transformation for bodily energy.

Thus, as a systemic cavitary entity inherent to TCM theory, the “waterway” function of the Sanjiao suggests a correspondence with the interstitium’s postulated role as a systemic fluid network. Its function of moving “Qi” and facilitating “Qi transformation” could be understood as a process regulating information and energy, which coincides with proposed mechanisms in the interstitial system where signaling molecules and exosomes may communicate over long distances via the fluid phase. Both concepts emphasize holistic connectivity, systematicity, and dynamic equilibrium.

The classification of the interstitium as an “organ” remains a subject of ongoing scholarly discussion, and that its relationship to the fascial system is not yet definitively resolved. A relevant question arises: Could the limited acceptance of the interstitium as an organ be related to its apparent lack of clear “boundaries”? An organ is the largest aggregate of two or more tissue types or one or more basic morphological units, primarily characterized by defined structural boundaries, intrinsic vascular networks, and neural innervation. In contrast, the interstitial network transcends the boundaries of individual organs, maintaining continuity within connective tissue layers and across tissue interfaces—including the dermis and subcutaneous tissue of the skin, as well as the submucosa and attached mesentery of the intestine—thereby forming structural continuities that extend across organ boundaries (Park and Woo, 2012; Cenaj et al., 2021). Dissection of human cadavers has further demonstrated that this continuity pervades large multi-organ regions of the body, encompassing the entire dermis and the fasciae of different organs and organ systems (van der Wal, 2015; Stecco et al., 2018).

### 2.4 Structure and function of fascial system: toward an integrative view beyond a “wrapping membrane”

#### 2.4.1 From static wrap to dynamic network

Research over the past two decades has substantially revised the traditional anatomical perception of fascia as merely a simple membranous structure wrapping muscles. Modern investigations suggest that the fascial system may represent a broadly distributed and functionally interconnected connective tissue network (Adstrum et al., 2017), composed of collagen, elastin, and glycosaminoglycans such as hyaluronan. Current evidence indicates that this system appears to envelop, permeate, and connect various structures—including organs, muscles, bones, nerves, and blood vessels—contributing to a dynamic environment potentially involved in bodily support and systemic communication (Adstrum et al., 2017).

#### 2.4.2 Structural layering: a multi-tiered system

Structurally, the fascial system can be described as a multi-layered system. Based on the consensus from the 2018 International Fascia Research Congress and subsequent studies (e.g., work by the Stecco family (Stecco et al., 2011) and Langevin’s team (Langevin et al., 2011)), it is often categorized into four anatomical organs: superficial fascia (subcutaneous, involved in metabolism and cushioning), musculoskeletal (deep) fascia (dense, primarily responsible for force transmission and separation), visceral fascia (which envelops and stabilizes internal organs), and neural fascia (protecting the central nervous system). Even specialized structures such as joint capsules and neurovascular sheaths are frequently included within the fascial family.

#### 2.4.3 Functional spectrum: beyond mechanical support

The functions of the fascial system appear to extend well beyond mechanical support. First, it is considered part of the body’s mechanical transmission and bio-informational regulatory system: fascia is rich in various receptors (e.g., nociceptors, mechanoreceptors); the epimysium within the deep fascia is thought to efficiently transmit muscle contraction force to tendons (Huijing, 2009). The fibroblasts within it seem capable of sensing mechanical stress and participating in local immune regulation and tissue repair through the release of cytokines (Langevin, 2021). It has been proposed as a potential interface for sensory information exchange between the body and the brain. Second, it forms important gliding interfaces, where the interstitial fluid within helps to ensure low-friction movement between tissues. In fact, this sophisticated modern understanding of the integrated fascial-interstitial system bears certain conceptual parallels to the ancient Chinese medical system’s progression from the exterior to the interior—encompassing Couli (interstitial spaces), Fenrou (intermuscular spaces), Xigu (larger grooves and joint spaces), and Moyuan (membrane source).

### 2.5 Structure and function of the TCM “mocou system”: toward a classical “cavity−channel” anatomy

In TCM theory, Couli (interstitial spaces of the skin and muscles), Fenrou (intermuscular spaces), Xigu (larger muscular grooves and joint spaces), and Moyuan (membrane source) are often understood as collectively forming a continuous “cavity−channel” system that extends from the body surface toward the internal organs, from the superficial to the deep layers. Their conventional locations and functions may be outlined as follows:

#### 2.5.1 Couli (interstitial spaces of the skin and muscles)

As noted in the Huangdi Neijing Suwen•Pibu Lun: “When pathogenic factors invade the skin, the Couli open; when they open, the pathogens enter and lodge in the connecting vessels.” (Daihua, 2005a) The Jinkui Yaolue•Zangfu Jingluo Xianhou Bingmai Zheng further elaborates: “Cou refers to the places where the Sanjiao communicates and converges with the genuine Qi (Yuanzhen), and where Qi and blood infuse; Li refers to the textures and patterns of the skin and internal organs.” (Ren, 2005) In common interpretation, Couli is generally regarded as referring to the textures, patterns, and interstitial gaps of the skin, muscles, and internal organs—the tissue architecture at the junction between skin and muscle. Its primary functions are commonly considered to include: serving as a major pathway for the circulation and nourishment of Defensive Qi (Weiqi) and for the distribution and excretion of fluids (e.g., sweat); acting as a gateway for the entry and exit of Yang Qi. Regarded as the body’s outermost defensive layer, it is thought to sense external climatic changes (such as cold, heat, dryness, and dampness) and to help regulate body temperature and fluid balance through mechanisms of opening and closing. It is often viewed as the first barrier against the invasion of external pathogens (e.g., wind−cold).

#### 2.5.2 Fenrou (intermuscular spaces)

As referenced in the Lingshu•Bencang, Fenrou generally denotes the gaps and boundaries between muscles, often described as pathways for the movement of Nutritive and Defensive Qi (Yingqi and Weiqi). The text states, “Defensive Qi is what warms the Fenrou, fills the skin, enriches the Couli, and governs opening and closing.” (Daihua, 2005b).

#### 2.5.3 Xigu (perivascular and perineural spaces, joint cavities, and deep compartments)

The Suwen•Qixue Lun notes, “The large gatherings of flesh are called Gu (valleys), the small gatherings are called Xi (streams). Between the separations of flesh, at the convergences of Xi and Gu, the Nutritive and Defensive Qi flow, and the great Qi converges.” (Daihua, 2005c) This description has often been compared to modern anatomical structures such as intermuscular grooves, joint cavities, bursae, perivascular and perineural spaces, and deep fascial compartments. Xigu are regarded as regions where Qi and blood may deeply accumulate and permeate, thereby contributing to the nourishment of tendons and bones and playing a role in joint stability and mobility.

#### 2.5.4 Moyuan (pleuro-diaphragmatic interspace/membrane source)

As discussed in Xue Shengbai’s Shire Tiaobian: “The Moyuan communicates externally with the muscles and internally approaches the stomach and intestines; it is considered the portal of the Sanjiao, and indeed may be regarded as occupying a half-exterior, half-interior position in the body. Pathogens received from above are said to head directly for the central pathway, and thus diseases are often described as returning to the Moyuan.” (Xue, 2007) It is commonly defined as the “gateway of the Sanjiao,” viewed as a pivotal junction between the interior and exterior that appears to serve as a hub for inward and outward passage. In the Wen Yi Lun (Treatise on Pestilence), Wu Youxing noted: “When pathogenic factors enter via the mouth and nose, their lodging is described as being neither purely within the viscera nor purely within the channels and collaterals. They are said to settle in the region adjacent to the spine, not far from the exterior and close to the stomach. This is regarded as the boundary between the exterior and interior—the so-called half-interior, half-exterior region—which the Suwen · Malaria Chapter refers to as ‘transversely connecting to the Moyuan (membrane-source) ‘.” (Youxing, 2007) This suggests its location in a half-interior, half-exterior space, “externally connecting to the muscles, and internally adjacent to the stomach and fu−organs.” Understood as a channel connecting the Upper, Middle, and Lower Burners, it is often seen as a key gateway for the movement and transportation of Original Qi, body fluids, and pathogenic factors. It is frequently portrayed as an initial site where the interaction between healthy qi and pathogenic factors takes place, potentially influencing the direction of disease transmission—whether inward or outward.

#### 2.5.5 Integration of the mocou system

**Figure 2:**
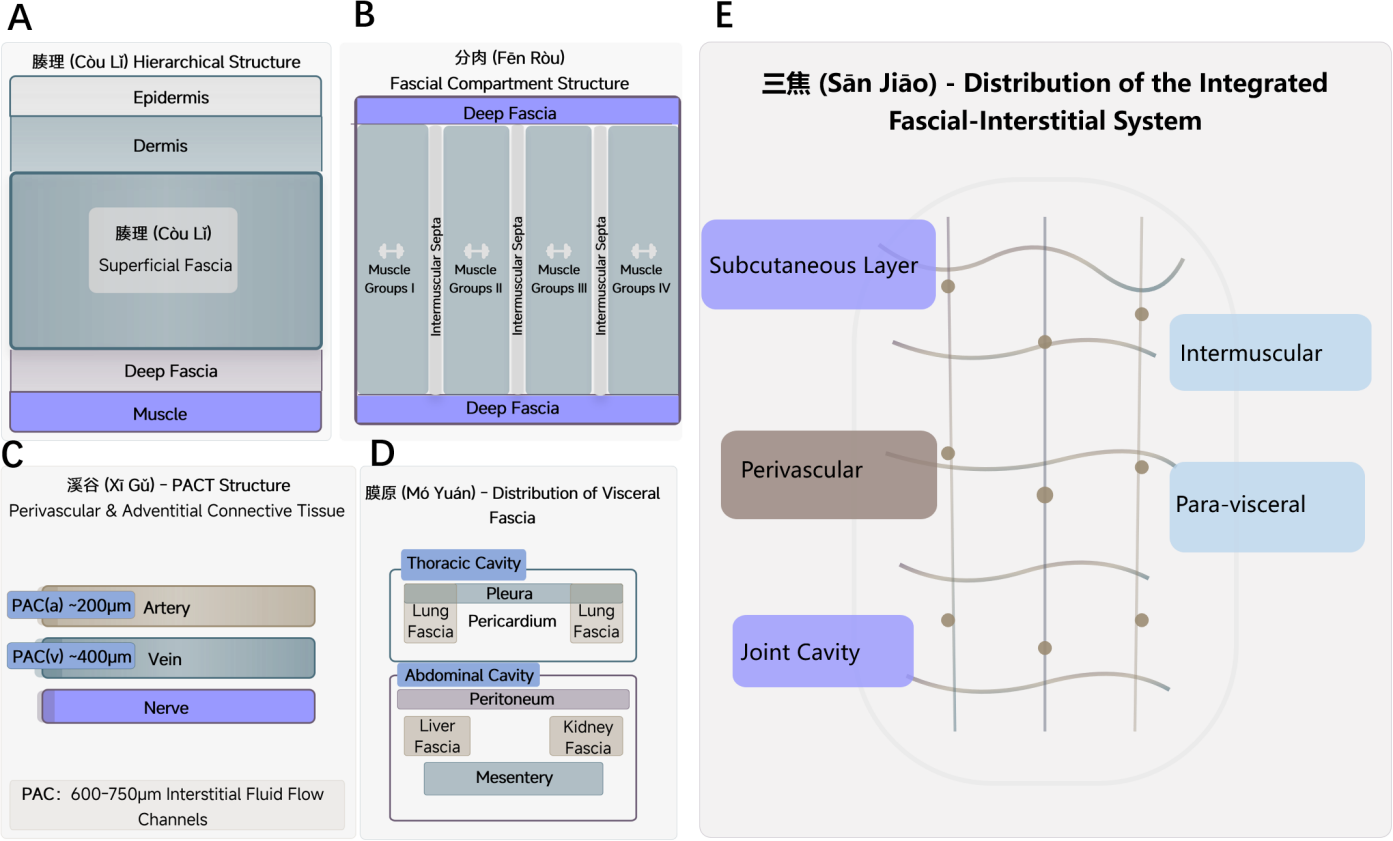
A conceptual schematic illustrating the proposed analogy between the TCM Membrane-Interstices (Sanjiao-Mocou) system and the modern integrated fascial-interstitial system. The figure depicts five progressive anatomical levels from superficial to deep, mapped onto a human silhouette. **(A)** Superficial Level (Interstices/Couli). A light-colored area beneath the skin represents the Interstices (Couli), which corresponds anatomically to the Superficial Fascia and its associated fascial interstitium. This layer constitutes the most superficial compartment of the proposed connective tissue network. **(B)** Muscular Level (Muscular Interlayers/Fenrou). A striped area between major muscle groups represents the Muscular Interlayers (Fenrou), which corresponds to the Deep Fascia and Intermuscular Septa. This layer organizes muscles into functional compartments and facilitates force transmission. **(C)** Deep Somatic Level (Valleys & Grooves/Xigu). The perivascular adventitial connective tissue (PACT) represents a specialized component of the integrated fascial-interstitial system. Around arteries, the PACT forms a fluid channel approximately 400 μm in width, while around veins it is approximately 200 μm. These spaces, together with adjacent connective tissue, can coalesce into larger interstitial fluid channels (600–750 μm) along neurovascular bundles, consistent with the TCM concept of Xigu as deep pathways for Qi and fluid. **(D)** Visceral Level (Membrane Source/Moyuan). A mesh-like pattern surrounding internal organs represents the Membrane Source (Moyuan), analogous to the Visceral Fascia and Body Cavities (e.g., peritoneal and pleural spaces). This layer suspends and invests the internal organs. **(E)** Systemic Integration (Sanjiao). A semi-transparent network of flowing lines overlying the entire body represents the Sanjiao, conceptualized as the Whole-body integrated fascial-interstitial Network. This overarching system is hypothesized to function as a continuous fluid and information Channel, integrating all levels from **(A–D)** into a dynamic, body-wide cavitary organ system for transport and signaling.

These four TCM concepts—Couli, Fenrou, Xigu, and Moyuan—are generally not viewed in isolation but are thought to form a functionally coherent, depth−layered network. Contemporary research on the integrated fascial-interstitial system may offer anatomical and physiological insights that resonate with this classical model, providing a modern perspective for its interpretation (**Table 1**). A conceptual synthesis of this layered correspondence is illustrated in **Figure 2**.

**Table 1:** Structural and functional comparison of the TCM “Mocou-Sanjiao System” and the Modern “Integrated Fascial-Interstitial System”.

Dimension of comparison	TCM “mocou-sanjiao” system	Modern “integrated fascial-interstitial” system	Corresponding relationship & explanation
Overall Systemic Positioning	“Mocou-Sanjiao” System: A continuous, body-wide “cavity-channel” system extending from the exterior to the interior, providing the structural and functional foundation for the movement of Qi, Blood, and Body Fluids, as well as information transmission.	“Integrated Fascial-Interstitial” System: A continuous body-wide mechanical-fluid network comprising collagenous layers (providing mechanical support) and hyaluronan-rich interstitial layers (enabling fluid flow and signal transmission).	Both represent a systemic, continuous, cavity-like network within the body, showing high consistency in their overall positioning.
Structural Layering (From Superficial to Deep)	1. Couli: Textures, patterns, and interstitial gaps of the skin, muscles, and internal organs (at the skin-muscle junction). 2. Fenrou: Gaps and boundaries between muscles. 3. Xigu: Larger intermuscular grooves, joint cavities, bursae, perivascular and perineural spaces, and deep fascial spaces. 4. Moyuan: Located in the half-exterior, half-interior space, “externally connecting to muscles, internally adjacent to the stomach and fu-organs,” considered the gateway of the Sanjiao.	1. Superficial Fascia: Subcutaneous layer, involved in metabolism and cushioning, containing fascial interstitium. 2. Deep Fascia: Dense connective tissue layers enveloping muscle groups and separating compartments, primarily responsible for force transmission. 3. Deep Compartments and Perivascular Spaces: Joint capsules, bursae, intermuscular septa, and perivascular/perineural spaces—all containing fascial interstitium. 4. Visceral Fasciae and Body Cavity Linings: Membranes enveloping and supporting internal organs, forming the parietal and visceral layers of serous cavities (e.g., pleural, peritoneal), including their associated fascial interstitium.	The four layers from superficial to deep show a layered correspondence in anatomical location and tissue characteristics.
Core Components	Qi, Blood, Body Fluids (the material and energetic substances circulating within the cavity-channels).	Collagen (Types I, III), Elastin, Glycosaminoglycans (e.g., Hyaluronan), Tissue Fluid (comprising the hyaluronan-rich interstitial fluid within the fascial interstitium).	TCM’s “Qi, Blood, Body Fluids” can be interpreted as a functional summarization of the interstitial fluid and its contained signaling molecules, nutrients, and metabolic products.
Primary Functions	1. Circulating Qi, Blood, & Fluids: Providing pathways for the distribution of Nutritive/Defensive Qi, Blood, and Body Fluids. 2. Defense Against Pathogens: Couli govern opening/closing, are where Defensive Qi circulates, acting as the first barrier against external pathogens. 3. Information Transmission & Qi Transformation: Facilitating informational communication and functional coordination among organs via Qi movement (Qi Hua). 4. Regulating Balance: Regulating body temperature and fluid balance through fluid metabolism (sweat, urine, etc.).	1. Fluid Transport & Signal Conduction: The integrated fascial-interstitial system acts as a “fluid superhighway,” transporting nutrients, waste, hormones, immune cells, and signaling molecules like exosomes. 2. Mechanical Support & Force Transmission: The collagenous layers of the fascial system form a mechanical skeleton, transmitting muscle contraction force and maintaining organ position and joint stability. 3. Immune Surveillance & Inflammatory Response: Interstitial fluid rapidly delivers immune cells to sites of infection/injury; fascial fibroblasts participate in local immune regulation. 4. Maintaining Internal Homeostasis: Interstitial fluid helps maintain osmotic pressure and pH balance; fascial gliding interfaces ensure low-friction movement between tissues.	High functional correspondence: “Circulating Qi, Blood, Fluids” corresponds to “Fluid Transport & Signal Conduction”; “Defense Against Pathogens” corresponds to “Immune Surveillance & Inflammatory Response”; “Information Transmission/Qi Transformation” corresponds to “Cellular Communication & Mechanosensing”; “Regulating Balance” corresponds to “Maintaining Internal Homeostasis.”
Key Hub/Gateway	Moyuan: Defined as the “gateway of the Sanjiao,” located at the half-exterior, half-interior level. It is a pivotal hub for the entry, exit, and transportation of pathogens, Original Qi (Yuanqi), and Body fluids, determining the direction of disease progression.	Fascial Compartments & Serous Cavities: Such as the pleural, peritoneal, and pericardial cavities, as well as potential spaces formed by fascia (e.g., retroperitoneal space). These are critical pathways and barriers for fluid accumulation, infection spread, and tumor metastasis.	Moyuan shares high similarity in location (half-exterior, half-interior) and function (gateway, hub, pathway for pathological transmission) with the anatomical and physiological features of serous cavities and deep fascial spaces.
Corresponding Pathological States	1. Waterway Obstruction: Impaired fluid metabolism, e.g., edema, phlegm-fluid retention. 2. Qi Dynamic Stagnation/Blockage: Impaired Qi movement, e.g., distension, pain. 3. Phlegm and Blood Stasis Intertwining: Accumulation of pathological products, forming masses, nodules, fibrosis.	1. Interstitial Fibrosis/Edema: Excessive collagen deposition or abnormal tissue fluid accumulation, leading to organ hardening or swelling. 2. Fascial Adhesion/Tightness: Loss of fascial glide, leading to pain, restricted movement, altered force lines. 3. Chronic Inflammation/Tumor Metastasis: Spread via interstitial-lymphatic channels, abnormal deposition and growth in the interstitial-fascial network.	Shared pathological mechanisms: TCM’s “Waterway Obstruction” corresponds to dysfunctional fluid dynamics due to interstitial edema or fibrosis; “Qi Dynamic Stagnation” corresponds to disordered mechanical environment and pain due to fascial tension and adhesion; “Phlegm and Blood Stasis Intertwining” corresponds to abnormal deposition and growth in the interstitial-fascial network, as seen in chronic inflammation, fibrosis, or tumors.
Systemic Integrative View	Sanjiao: As the “solitary Fu-organ,” it commands the Qi transformation and water metabolism of the entire body. It is the functional generalization of the Mocou System, emphasizing the integrated movement and systemic coordination of Qi, Blood, and Water.	Integrated Fascial-Interstitial System: The collagenous layers as the structural framework and the hyaluronan-rich interstitial layers as the fluid-filled spaces together constitute a ”systemic cavitary network” that provides both mechanical support and is responsible for fluid circulation and information transmission.	The Sanjiao as the functional commander aligns perfectly with the Integrated Fascial-Interstitial System as a unified structural-functional entity in their systemic, holistic concept. Together, they describe a continuous cavitary network system within the human body that is neither a solid organ nor a mere empty space, but one with significant physiological functions.

This table is based on the detailed discussion in the main text regarding the TCM “Mocou-Sanjiao” system and the modern “Integrated Fascial-Interstitial” system. It provides a systematic comparison across multiple dimensions—structure, function, and pathology—to visually demonstrate the high degree of analogy and complementarity between these two theoretical frameworks in describing what may be the same foundational structural-functional system in the human body.

## 3 Conclusion & significance

Based on the preceding analysis, several key points can be summarized:

First, a structural and functional correspondence appears to exist between these systems. The distribution of the interstitium in Western medicine and its proposed role in facilitating fluid transport seem to align with the TCM concept of the Sanjiao as the “waterway” and the “commander of Qi transformation.” Similarly, the multi-level classification of the fascial system (superficial, deep, visceral) and its mechanical and informational regulatory functions may parallel the layered “cavity-channel” system of TCM, comprising Couli, Fenrou, Xigu, and Moyuan.

Second, the integrated fascial-interstitial system may exhibit systemic unity. Within this unified system, fascia and the fascial interstitium can be viewed as complementary components of a broader connective tissue matrix. The collagenous layers of fascia might provide a dense mechanical framework—analogous to “walls and skeleton”—that establishes macroscopic anatomical compartments. In contrast, the hyaluronan-rich fascial interstitium could be understood as representing the looser, fluid-rich compartmental interiors—akin to interconnected “rooms and corridors”—facilitating fluid dynamics and cellular communication.

Third, the TCM Mocou-Sanjiao system and the Western fascial-interstitial system complex may together offer a potential anatomical and physiological basis for understanding meridians. While the objective nature of meridians remains under investigation—with some viewing them as a “supra-anatomical” functional network—the anatomical continuity within the integrated fascial-interstitial system offers a plausible model. This integrated system could be conceptualized as a continuous, cavity-rich matrix contributing to mechanical support, fluid circulation, cellular signaling, and energy/information transfer. This perspective appears consistent with classical TCM descriptions of meridian function, such as those found in the Lingshu·Hailun (“The twelve regular meridians internally connect to the Zang-Fu organs and externally network with the limbs and joints…”) and the Nanjing (“The meridians move Qi and blood, connect Yin and Yang, to nourish the body”). Consequently, pathological conditions like fascial adhesion or interstitial fibrosis may correspond to TCM disease mechanisms involving meridian dysfunction, including “waterway obstruction,” “Qi dynamic stagnation,” or “phlegm-blood stasis.”

The analogy hypothesis outlined here may offer an integrative framework bridging historical and modern perspectives on human physiology. We acknowledge that the conceptualization of the fascia and interstitium as distinct organs—or as components of an integrated organ system—remains an evolving and unsettled area of anatomical discourse. These concepts emerged primarily following the 2018 publication by Benias et al. and subsequent investigations into the systemic nature of interstitial spaces. However, we suggest that the very fluidity and openness of these contemporary anatomical models—their departure from rigid, pre-existing categorical boundaries, pre-existing categorical boundaries—may make them well suited to serve as a conceptual bridge between Western anatomical science and the holistic, systems-based framework of TCM. This framing appropriately positions the discussion within the context of emerging knowledge rather than settled fact. The hypothesis may also provide a theoretical foundation for future interdisciplinary research—such as exploring mechanisms underlying acupuncture and manual therapies, investigating the pathophysiology of chronic conditions (e.g., musculoskeletal disorders, hypertension, diabetes), and informing novel therapeutic strategies focused on fascial and interstitial health.
